# Satellite DNAs Unveil Clues about the Ancestry and Composition of B Chromosomes in Three Grasshopper Species

**DOI:** 10.3390/genes9110523

**Published:** 2018-10-26

**Authors:** Diogo Milani, Vanessa B. Bardella, Ana B. S. M. Ferretti, Octavio M. Palacios-Gimenez, Adriana de S. Melo, Rita C. Moura, Vilma Loreto, Hojun Song, Diogo C. Cabral-de-Mello

**Affiliations:** 1 Instituto de Biociências/IB, Departamento de Biologia, UNESP—Universidade Estadual Paulista, Rio Claro, São Paulo 01049-010, Brazil; azafta@gmail.com (D.M.); vbbardella@gmail.com (V.B.B.); anabeatrizferretti@gmail.com (A.B.S.M.F.); octavio.palacios@ebc.uu.se (O.M.P.-G.); 2 Department of Evolutionary Biology, Evolutionary Biology Center, Uppsala University, 75236 Uppsala, Sweden; 3 Instituto de Ciências Biológicas, Laboratório de Biodiversidade e Genética de Insetos, UPE—Universidade de Pernambuco, Recife 50100-130, Pernambuco, Brazil; adrianadesouzamelo@gmail.com (A.d.S.M.); ritamoura.upe@gmail.com (R.C.M.); 4 Centro de Biociências/CB, Departamento de Genética, UFPE—Universidade Federal de Pernambuco, Recife 50670-901, Pernambuco, Brazil; vloreto@bol.com.br; 5 Department of Entomology, Texas A&M University, 2475 TAMU, College Station, TX 77843-2475, USA; hsong@tamu.edu

**Keywords:** fluorescent in situ hybridization, Orthoptera, satellite DNA, supernumerary chromosome, RepeatExplorer

## Abstract

Supernumerary (B) chromosomes are dispensable genomic elements occurring frequently among grasshoppers. Most B chromosomes are enriched with repetitive DNAs, including satellite DNAs (satDNAs) that could be implicated in their evolution. Although studied in some species, the specific ancestry of B chromosomes is difficult to ascertain and it was determined in only a few examples. Here we used bioinformatics and cytogenetics to characterize the composition and putative ancestry of B chromosomes in three grasshopper species, *Rhammatocerus brasiliensis*, *Schistocerca rubiginosa*, and *Xyleus discoideus angulatus*. Using the RepeatExplorer pipeline we searched for the most abundant satDNAs in Illumina sequenced reads, and then we generated probes used in fluorescent in situ hybridization (FISH) to determine chromosomal position. We used this information to infer ancestry and the events that likely occurred at the origin of B chromosomes. We found twelve, nine, and eighteen satDNA families in the genomes of *R. brasiliensis*, *S. rubiginosa*, and *X. d. angulatus*, respectively. Some satDNAs revealed clustered organization on A and B chromosomes varying in number of sites and position along chromosomes. We did not find specific satDNA occurring in the B chromosome. The satDNAs shared among A and B chromosomes support the idea of putative intraspecific ancestry from small autosomes in the three species, i.e., pair S11 in *R. brasiliensis*, pair S9 in *S. rubiginosa*, and pair S10 in *X. d. angulatus*. The possibility of involvement of other chromosomal pairs in B chromosome origin is also hypothesized. Finally, we discussed particular aspects in composition, origin, and evolution of the B chromosome for each species.

## 1. Introduction

Eukaryotic genomes exhibit repetitive DNA sequences including noncoding tandemly repeated satellite DNA (satDNA). These sequences exhibit extensive variability in copy number and nucleotide sequence, even among phylogenetically related species. Arrays of satDNAs are usually located in the centromeric and telomeric heterochromatin of the chromosomes, although they have also been reported in the euchromatic region. Furthermore, satDNAs are frequently enriched on sex chromosomes and supernumerary (B) chromosomes, as they are greatly enriched in heterochromatin [1,2,3].

Supernumerary B chromosomes occur in approximately 15% of eukaryotes as dispensable elements (i.e., not required for normal organismal development), frequently heterochromatic and enriched repetitive DNAs, including the satDNAs, which can have implications for B chromosome evolution. Generally, B chromosomes do not recombine with A chromosomes (normal complement), and B chromosome sequences evolve at a higher evolutionary rate than A elements [4,5]. Since the first discovery of the B chromosome [6], the specific ancestry of the studied B chromosomes in eukaryotes has remained largely unknown. For decades, repetitive DNAs have been used to try to ascertain the ancestry and to describe the B chromosome composition in some species. In that way, satDNAs have helped the understanding of the evolutionary history of B chromosomes with intraspecific (from host genome) or interspecific (resultant of species hybridization) origin, for example, in grasshoppers [7,8], wasps [9], fish [10], and plants [11], among others.

Among the grasshoppers, approximately 12% of the species harbor B chromosomes. Some families seem to be hotspots for B chromosome presence, such as Acrididae, with 17.1% of the species harboring B chromosomes, unlike Romaleidae in which only 4% of the species is harboring B chromosomes [12]. As generally observed in eukaryotes, some repetitive DNAs populate the B chromosomes of grasshoppers, like the multigene families for rDNAs [13,14], histone genes [14,15], and U snDNA [16], transposable elements [8,17,18], microsatellites [19], and satDNAs [7,8,13,20]. These sequences shed light on B chromosome composition, variability, and evolutionary dynamics. Concerning satDNAs, their presence in the B chromosomes of grasshoppers is known only in a few species, including *Locusta migratoria* [8], *Abracris flavolineata* [20], *Eyprepocnemis plorans* [13], and *Eumigus monticola* [7].

The search for satDNAs in genomic data was facilitated more recently by analyzing reads from next generation sequencing (NGS) using bioinformatics approaches, like RepeatExplorer software [21]. RepeatExplorer has been a useful tool for detecting satDNAs for probe generation and chromosome mapping in species with B chromosomes, helping to unveil the composition and abundance of satDNAs in those chromosomes as well as their relationships with A elements as well [7,8,11,22].

By combining genomics and cytogenetics, we aimed to elucidate the genome content of satDNAs and used this information to track the possible ancestry of B chromosomes in three grasshopper species, *Rhammatocerus brasiliensis* (Acrididae: Gomphocerinae), *Schistocerca rubiginosa* (Acrididae: Cyrtacanthacridinae), and *Xyleus discoideus angulatus* (Romaleidae: Romaleinae) belonging to two families. The family Acrididae, which is currently most diverse lineage within the orthopteran suborder Caelifera, diverged from its sister lineage, which includes the family Romaleidae, in the late Cretaceous (~78 mya, million years ago) based on a fossil-calibrated divergence time estimate. Two acridid subfamilies included in this study, Gomphocerinae and Cyrtacanthacridinae, each belong to different clades within the family, and they are estimated to have diverged in the late Eocene [23]. For this purpose, we first made a prediction of the most abundant satDNAs in genomes by using the RepeatExplorer tool. Then we recovered the fragments by PCR and designed probes of each satDNA of the three species to use in fluorescent in situ hybridization (FISH) experiments. This allows for the investigation of spatial patterns of satDNAs that have preferentially accumulated in B chromosomes compared to autosomes. We found distinct patterns of satDNA distribution on B chromosomes that are shared with some A chromosomes or with exclusive A chromosomes. Based on these data, it is possible to hypothesize the ancestry of the B chromosome from small autosomes and to discuss aspects of the evolution of B chromosomes in the three species.

## 2. Material and Methods 

### 2.1. Animal Sampling, Chromosome Preparations, and Genomic DNA Sequencing

Adult animals of *R. brasiliensis* were collected at 07°45′00′′ S; 34°05′10′′ W Ilha de Itamaracá/PE (Brazil) and 07°50′56′′ S 35°19′14′′ W Lagoa do Carro/PE (Brazil); *X. d. angulatus* at 07°12′47′′ S 39°18′55′′ W Juazeiro do Norte/CE (Brazil); *S. rubiginosa* at 29°25.908′ N 82°24.060′ W Levy County/Florida (USA). Testes were fixed with Carnoy’s modified solution (3:1, 100% Ethanol:Glacial Acetic Acid) and stored at −20 °C until use for slides preparation. Femurs were immersed in 100% ethanol and stored at −20 °C for genomic DNA (gDNA) extraction.

The genomic DNA sequencing method for the *S. rubiginosa* (male) specimen was previously described [24]. For the *R. brasiliensis* (female) and *X. d. angulatus* (male) specimens DNA extraction was performed using the phenol/chloroform-based procedure described previously [25]. Sequencing was conducted by the Illumina company (Inc., San Diego, CA, USA) with a HiSeq 4000 to obtain paired-ends libraries (2 × 101 bp) using the service of Macrogen Inc. (Seoul, Republic of Korea).

We applied conventional staining with 5% Giemsa for chromosome observation and identification of individuals harboring B chromosomes. The C-banding for heterochromatin identification was performed according to a previously described method [26].

### 2.2. SatDNAs Searching by Graph-Based Clustering Method

Prior to RepeatExplorer graph-based clustering analysis, we preprocessed and checked the quality of the paired-ends reads of each species using FastQC [27]. Preprocessing of the reads was performed following default parameters using the public online platform: https://repeatexplorer-elixir.cerit-sc.cz/galaxy/. Reads were processed with a “quality trimming tool”, “FASTQ interlacer” on paired end reads, “FASTQ to FASTA” converter, and “RepeatExplorer clustering” all with default recommended options [21]. First we searched and selected, by visual observation, the clusters that showed high graph density that indicated proximity with satDNAs families [28]. Contigs from the selected clusters were deeply explored and manually searched for sequences with tandem pattern confirmed by the dot plot graphics implemented using Geneious v4.8.5 software [29]. The consensus monomer of each satDNA family of each species was used as the query in two databanks, BLAST (http://www.ncbi.nlm.gov/Blast/) and Repbase (http://www.girinst.org/repbase/), to check similarity with another sequence deposited and described. Abundance of each satDNA family was calculated with the number of reads of each cluster divided by the total number of reads used in the “RepeatExplorer clustering” protocol [21]. Nucleotide divergence was calculated using the RepeatMasker package with specific parameters provided in the scripts program protocol to calculate Kimura divergence values [30]. Superfamilies (SF) were considered by comparing consensus monomer of each satDNA against all of them from each species independently using the Geneious v4.8.5 software [29] assembly tool, alternating overlap identity following the same considerations as a previous work [31]. We classified each identified satDNA family according to a previous method [31], considering the species name abbreviation and decreasing abundance, followed by the consensus monomer size; they were numbered in decreasing order of abundance. The sequences were deposited in GenBank under the accession numbers MH900339–MH900377.

### 2.3. Amplification of SatDNAs through PCR, Probes and Fluorescence In Situ Hybridization

We used the consensus sequences of each satDNA family of each species to design divergent primers manually or using the Primer3 tool [32] implemented in Geneious v4.8.5 software [29] (Supplementary Table S1). Polymerase chain reactions (PCRs) were performed using 10× PCR Rxn Buffer, 0.2 mM MgCl_2_, 0.16 mM dNTPs, 2 mM of each primer, 1 U of *Taq* Platinum DNA Polymerase (Invitrogen, San Diego, CA, USA), and 50–100 ng/μL of template DNA. The PCR conditions included an initial denaturation at 94 °C for 5 min and 30 cycles at 94 °C (30 s), 55 °C (30 s), and 72 °C (80 s), plus a final extension at 72 °C for 5 min. The PCR products were visualized on a 1% electrophoresis agarose gel. The monomeric bands were isolated and purified using the Zymoclean™ Gel DNA Recovery Kit (Zymo Research Corp., The Epigenetics Company, CA, USA) according to the manufacturer’s recommendations and then used as template for reamplification using the same PCR conditions. The monomers were sequenced by the Sanger method using the service of Macrogen Inc. to confirm the amplification of desired sequence.

FISH was performed in meiotic chromosomes using one or two probes according to a method described previously [33] with some adjustments as outlined previously [34]. The probes labeled with digoxigenin-11-dUTP were detected using anti-digoxigenin rhodamine (Roche, Mannheim, Germany), and probes labeled with biotin-14-dATP were detected using Streptavidin Alexa Fluor 488-conjugated (Invitrogen, San Diego, CA, USA). The preparations were counterstained using 4′,6-diamidine-2′-phenylindole (DAPI) and mounted in VECTASHIELD (Vector, Burlingame, CA, USA). FISH results were observed using an Olympus microscope BX61 (Tokyo, Japan) equipped with a fluorescent lamp and the proper filters. Images were obtained using a DP71 cooled digital camera in grayscale and then pseudo-colored in blue for chromosomes and red or green for hybridization signals, merged and optimized for brightness and contrast using Adobe Photoshop CS6. To describe the patterns of satDNA chromosomal distribution distinct cells were analyzed, including diplotene, metaphase I, metaphase II, and mitotic metaphase.

## 3. Results

### 3.1. Karyotypes, B Chromosomes, and Heterochromatin Distribution

Occurrence of a karyotype consisting of 2n = 23,X0, and presence of B chromosomes observed here for *R. brasiliensis* and *X. d. angulatus* were previously reported by different authors [14,35], including in the same population, i.e., Juazeiro do Norte/CE for *X. d. angulatus* [36]. We report for the first time the presence of B chromosomes in *R. brasiliensis* from Lagoa do Carro/PE. The karyotype of *S. rubiginosa*, described here for the first time, is also 2n = 23,X0 as observed for other species from the same genus, like *S. gregaria* [37], *S. pallens*, and *S. flavofasciata* [38]. Among the five individuals of *S. rubiginosa,* two presented B chromosomes. We classified autosomal chromosomes of the three species in three distinct groups considering size: three long chromosomes (L1–L3), five medium (M4–M8), and three small (S9–S11). 

**Figure 1:**
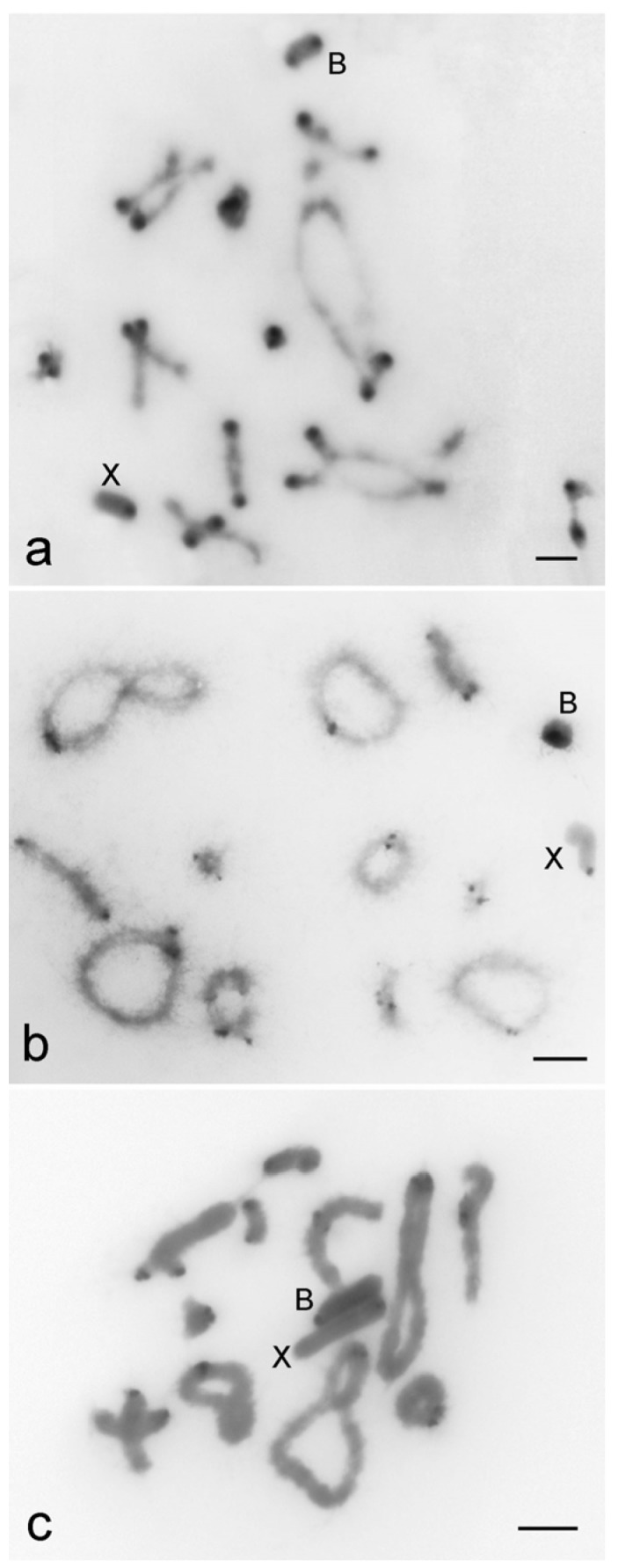
C-banding revealing the heterochromatin location in diplotene chromosomes of *Rhammatocerus brasiliensis* (**a**), *Schistocerca rubiginosa* (**b**), and *Xyleus discoideus angulatus* (**c**). Note the pericentromeric location of C-positive blocks on A chromosomes and the distinct patterns for the B chromosome of the three species, i.e., pericentromeric and distal in *R. brasiliensis*; pericentromeric and interstitial (close to the centromere) in *S. rubiginosa*; along the entire chromosome with darker band in pericentromeric region in *X. d. angulatus*. The X and B chromosomes are indicated. Bar = 5 μm.

The B chromosomes of the three species are acrocentric with variable pattern of heterochromatin distribution (Figure 1). For *R. brasiliensis* pericentromeric and distal blocks were observed (Figure 1a) and for *S. rubiginona* pericentromeric and interstitial blocks, close to the centromere, were noticed (Figure 1b). In *X. d. angulatus* the B chromosome was completely heterochromatic with deeper staining in the pericentromeric region (Figure 1c). Heterochromatin blocks restricted to pericentromeric areas were noticed for A chromosomes (Figure 1).

### 3.2. In Silico SatDNA Analysis

By using RepeatExplorer we predicted the most abundant satDNAs as follows, twelve, nine, and eighteen satDNA families in *R. brasiliensis*, *S. rubiginosa*, and *X. d. angulatus*, respectively. Monomer lengths varied from 36 to 410 nt in *R. brasiliensis*, from 107 to 441 nt in *S. rubiginosa*, and from 8 to 289 in *X. d. angulatus*. The predominance of families with monomer length higher than 100 nt was noticeable. Only for *X. d. angulatus*, satDNA families with monomer length smaller than 50 nt was observed (Table 1). Sequence similarity analysis revealed the presence of two similar satDNAs families in the genome of *R. brasiliensis*, RbrSat01-171 and RbrSat04-168 (superfamily SF1), In *X. d. angulatus* two superfamilies were noticed each composed by two satDNA families, SF1 (XanSat05-267 and XanSat07-279) and SF2 (XanSat09-130 and XanSat14-128). No similarity between satDNAs was noticed between species.

A+T content was variable from 44.5% to 63.9% (mean 57.63%) in *R. brasiliensis*, from 48.9% to 61.7% (mean 55.12%) in *S. rubiginosa*, and from 28.6% to 76.2% in *X. d. angulatus* (mean 59.15%). Predominance of A+T-rich satDNA families was observed, ten in *R. brasiliensis*, eight in *S. rubiginosa*, and fifteen in *X. d. andgulatus*. Concerning total abundance, the satDNAs represented 1.499% of the genome of *R. brasiliensis*, 2.172% of *S. rubiginosa*, and 2.322% of *X. d. angulatus* genomes. In all species, even the most abundant satDNA family represented less than 1% of the genome, i.e., 0.766% in *R. brasiliensis*, 0.73% in *S. rubiginosa*, and 0.627% in *X. d. angulatus*. The lowest abundance satDNA in *R. brasiliensis* corresponded to 0.01% of the genome, in *S. rubiginosa* to 0.026%, and in *X. d. angulatus* to 0.013% (Table 1).

### 3.3. Chromosomal Location of SatDNAs

**Figure 2:**
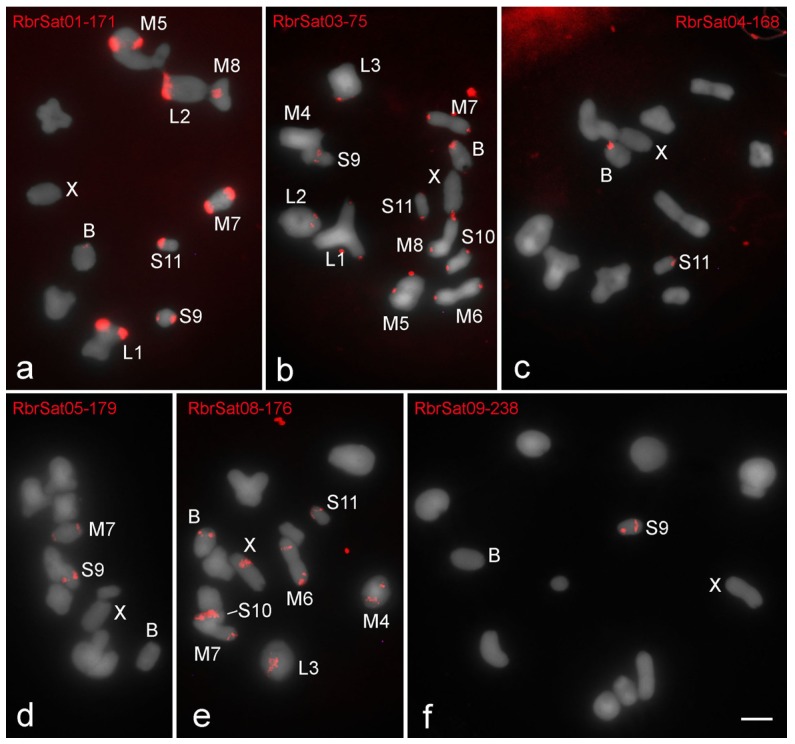
Fluorescent in situ hybridization (FISH) mapping on metaphase I of satDNAs identified in the genome of *R. brasiliensis*. The distinct satDNAs families are indicated. Chromosomes with signals, X and B chromosomes are identified. Note the presence on B chromosome of signals for (**a**) RbrSat01-171 (shared with some A chromosomes), (**b**) RbrSat03-36 (shared with all A chromosomes), (**c**) RbrSat04-168 (shared exclusively with pair S11), and (**e**) RbrSat08-176 (shared with some A chromosomes). For satDNAs (**d**) RbrSat05-179 and (**f**) RbrSat09-238 no signals were observed in B chromosome. Bar = 5 μm.

**Figure 3:**
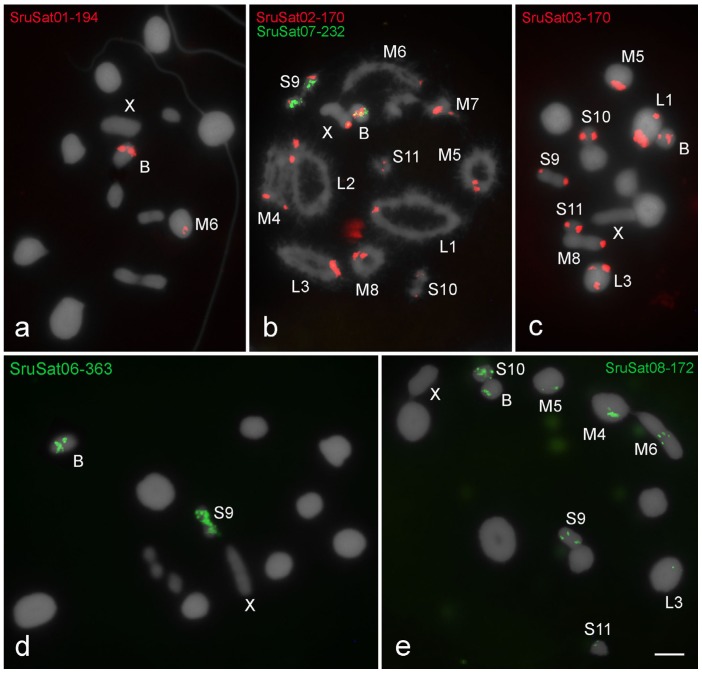
Chromosomal distribution of satDNAs in meiotic cells of *S. rubiginosa* (**a**,**c**,**d**,**e**) metaphase I and (**b**) diplotene. The distinct satDNAs families are indicated. Chromosomes with signals: X and B chromosomes are identified. Observe that the B chromosome harbors all satDNAs families, three of them shared with some A chromosomes (SruSat02-170, SruSat03-170, and SruSat08-172), and three exclusively shared with pair M6 (SruSat01-194) or pair S9 (SruSat06-363 and SruSat07-232). Bar = 5 μm.

**Figure 4:**
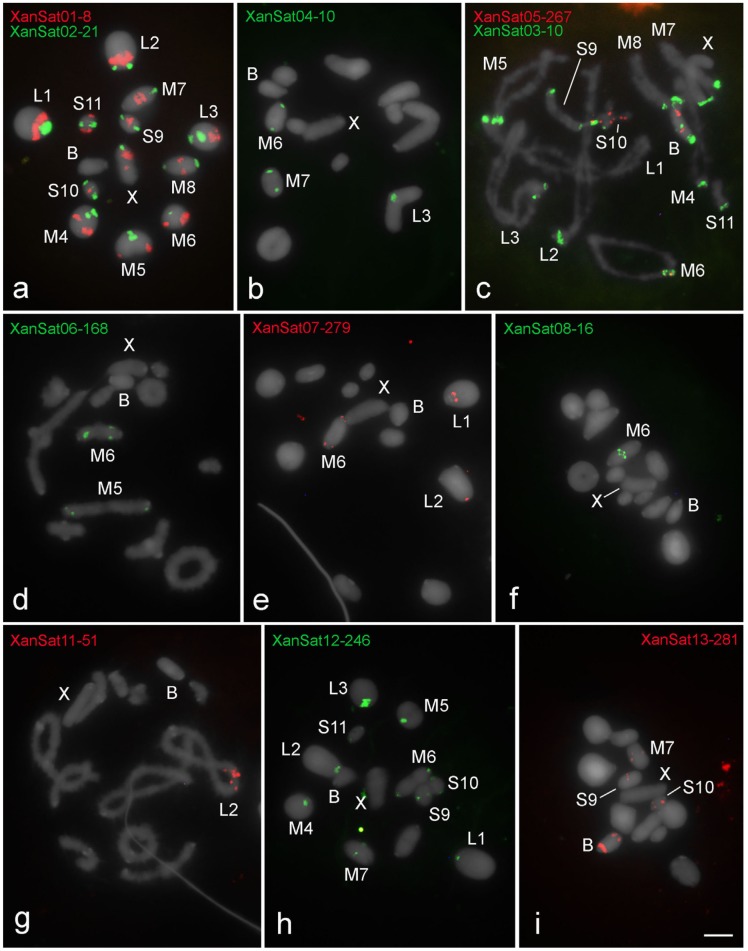
Patterns of chromosomal location revealed by FISH of eleven clustered satDNAs in *X. d. angulatus*. (**a**,**b**,**d**–**f**,**h**,**i**) metaphase I, (**c**,**g**) diplotene. The distinct satDNAs families are indicated. Chromosomes with signals: X and B chromosomes are identified. Note only three satDNA families on the B chromosome, XanSat03-10 (**c**), XanSat05-267 (**c**) and XanSat13-281 (**i**), all of which shared the pair S10. Multiply satDNA sites are observed for all satDNAs (**a**–**e**,**h**,**i**), except XanSat-08-16 (**f**) and XanSat11-51 (**g**). Bar = 5 μm.

All satDNA families recognized by RepeatExplorer analysis were accurately amplified by PCR and sequenced; FISH mapping showed signals for most of them (Table 2; Figure 2, Figure 3 and Figure 4). Six satDNA families revealed signals for *R. brasiliensis* (Figure 2) and *S. rubiginosa* (Figure 3), and for *X. d. angulatus* eleven satDNA families allowed identification of specific marks by FISH (Figure 4), representing clustered satDNAs. For the remaining satDNA families, six for *R. brasiliensis*, three for *S. rubiginosa,* and seven for *X. d. angulatus*, were nonclustered with no FISH signals (Table 2).

The distinct clustered satDNA families were variable in number and position within A chromosomes (Figure 2, Figure 3 and Figure 4). Only one satDNA was located exclusively on pericentromeric regions of A chromosomes in each species, RbrSat03-36 in *R. brasiliensis* (Figure 2b), SruSat02-170 in *S. rubiginosa* (Figure 3b), and XanSat03-10 in *X. d. angulatus* (Figure 4c). Heterochromatin blocks, like centromeres, were enriched in most satDNAs, but we also noticed a few satDNAs placed on the euchromatin of some chromosomes of *R. brasiliensis*: RbrSat08-176 (Figure 2e) and RbrSat09-238 (Figure 2f), and *S. rubiginosa*: SruSat03-170 (Figure 3c), SruSat06-363 (Figure 3d), SruSat07-232 (Figure 3b), and SruSat08-172 (Figure 3e). We observed that satDNA was more frequently distributed within the interstitial and distal euchromatin of *X. d. angulatus* (Figure 4a,c–g,i). The bias for pericentromeric position of satDNA was noticed by comparing the number of pericentromeric blocks with interstitial and distal ones for each species: *R. brasiliensis* twenty-eight pericentromeric and three interstitial; *S. rubiginosa* twenty-five pericentromeric, four interstitial, and five distal; *X. d. angulatus* forty-two pericentromeric, ten interstitial, and twelve distal (Table 2).

SatDNA unique to a specific A chromosome was a rare condition in the three species. It was noticed in *R. brasiliensis* for RbrSat04-168 (Figure 2c) and RbrSat09-238 (Figure 2f) in pair S11 and S9, respectively; SruSat01-194 (Figure 3a) in pair 6, and SruSat06-363 (Figure 3d) and SruSat07-232 (Figure 3b) in pair S9 of *S. rubiginosa*; in *X. d. angulatus* the repeats XanSat08-16 (Figure 4f) and XanSat11-51 (Figure 4g) were exclusive from the pairs M6 and L2, respectively. The number of satDNAs per specific A chromosome varied from 2 to 4 (mean 2.5) in *R. brasiliensis*, from 1 to 5 (mean 2.42) in *S. rubiginosa*, and from 3 to 9 (mean 5.08) in *X. d. angulatus*.

**Figure 5:**
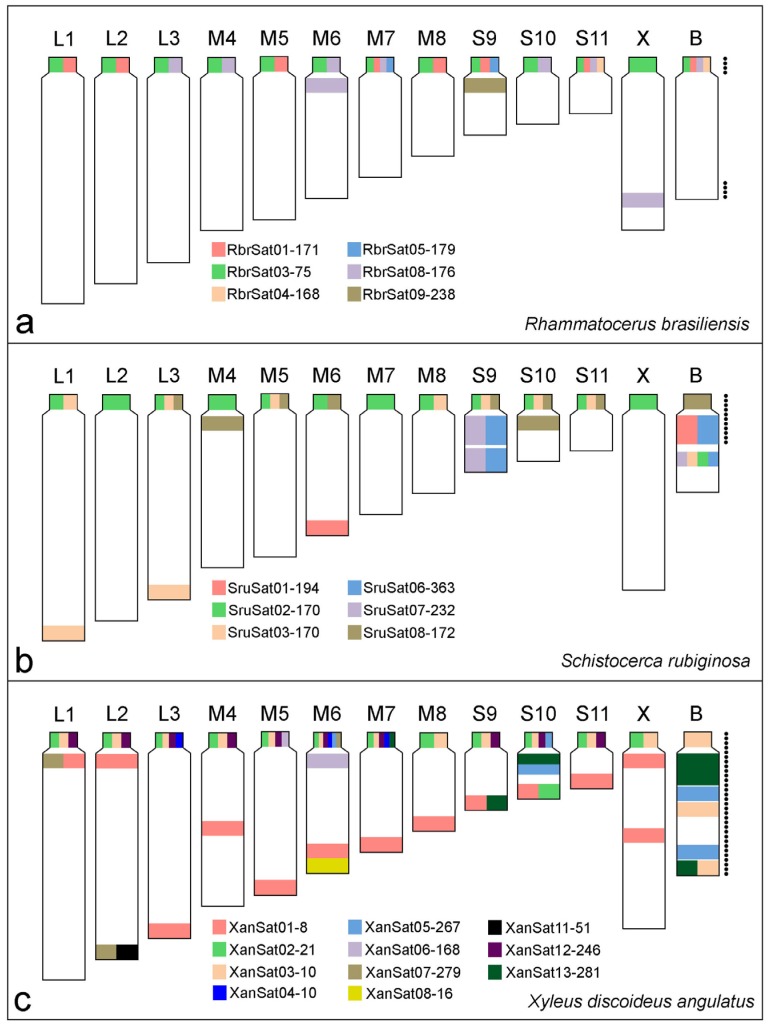
Ideograms summarizing the chromosomal location of clustered satDNAs in the three species of grasshoppers, (**a**) *R. brasiliensis*, (**b**) *S. rubiginosa*, and (**c**) *X. d. angulatus*. Each satDNA family is represented by one color. Black dots next to the B chromosomes indicate heterochromatin distribution in these chromosomes.

The B chromosomes were enriched with distinct satDNA families. All of them were shared with the A chromosomes but show distinct patterns of distribution, such as occurrence in multiple chromosomes or occurrence restricted to one or few elements (Figure 2, Figure 3, Figure 4 and Figure 5). Four satDNAs occupying pericentromeric regions were seen in the B chromosome of *R. brasiliensis*, RbrSat01-171 (Figure 2a), RbrSat03-36 (Figure 2b), RbrSat04-168 (Figure 2c), and RbrSat08-176 (Figure 2e). These satDNAs were shared with the chromosome S11, which accumulated them in the pericentromeric region. The satDNA RbrSat04-168 was exclusively shared between pair S11 and the B chromosome, while the others were also located in other chromosomes (Figure 2a–c,e and Figure 5a).

The B chromosome of *S. rubiginosa* harbored the six satDNAs that were found clustered in A chromosomes (Figure 3 and Figure 5b). SruSat08-172 (Figure 3e) was located in the pericentromeric region, while the other satellites were interstitially located presenting differences in signal size (Figure 3a–d). The chromosome S9 harbored five of the six satDNAs present in the B chromosome, and among them two were exclusive of pair S9 and B chromosome, SruSat06-363 (Figure 3d) and SruSat07-232 (Figure 3b). This was the chromosome with highest number of satDNAs shared with the B chromosome. SruSat01-194 was also in the B chromosome but among the A chromosomes this repeat was only in pair 6 (Figure 3a).

Among the eleven repeats mapped by FISH in *X. d. angulatus* chromosomes only three were visualized in the B chromosome, XanSat03-10 (Figure 4c), XanSat05-267 (Figure 4c), and XanSat13-281 (Figure 4i). For these repeats more than one signal was seen in the B chromosome (Figure 4c,i and Figure 5c). Xansat03-10 was located in pericentromeric, interstitial, and distal regions (Figure 4c and Figure 5c), XanSat05-267 presented two interstitial blocks (Figure 4c and Figure 5c) and XanSat13-281 was placed in interstitial and distal areas (Figure 4i and Figure 5c). We observed that none of the satDNAs located on the B chromosome were restricted to one A chromosome, XanSat03-10 (Figure 4c and Figure 5c) was located in all pericentromeric regions, XanSat05-267 (Figure 4c and Figure 5c) was located in pairs M6 and S10, and XanSat13-281 (Figure 4i and Figure 5c) was located in pairs M7, S9, and S10. Chromosome S10 shared the highest amount of satDNAs with the B chromosome (Figure 4c,i and Figure 5c).

## 4. Discussion

Appling bioinformatic and molecular cytogenetic approaches to determine the content of satDNA allowed for a rapid increase in characterization of these kinds of elements among Orthoptera. High-throughput analysis has allowed the characterization of 234 satDNAs families in seven species [7,31,39,40,41,42]. Here we describe for first time the chromosomal organization of the most abundant satDNAs populating the genomes of three grasshopper species from the Romaleidae and Acrididae families. They correspond to a total 39 satDNAs families contributing to the knowledge of chromosomal organization of this kind of repeat on B chromosome.

### SatDNAs Reveal Clues about B Chromosome Composition and Ancestry

Even though B chromosomes have been studied for a long time, we know very little about their ancestry in most species. B chromosome ancestry is known only in a few species [7], and therefore its origin still remains intriguing due in part to their high evolutionary rate. In grasshoppers, repetitive DNAs have been used to track B chromosome origin and composition in relatively few species. In *E. monticola* for instance, B chromosome ancestry is attributed to the autosomal pair S8 based on satDNAs analysis [7]. In *A. flavolineata*, the origin of B chromosome from pair 1 is attributed to the unique presence of U2 snDNA genes in these two chromosomes [16]. The ancestry of B chromosome in *L. migratoria* is related to pairs 8 and 9 due to the presence of satDNAs and histone genes in those chromosomes [8,15]. Previous works discussed the composition and putative origin of B chromosomes in two grasshoppers species studied here, *R. brasiliensis* and *X. d. angulatus*. However, it was not possible to elucidate a specific ancestral chromosome (see below). Based on satDNA content and organization we provide some clues about the ancestry of B chromosomes in these species, and additionally in *S. rubiginosa.*

The B chromosome of *R. brasiliensis* harbors four satDNAs, three of them are shared with multiple chromosomes (i.e., RbrSat01-171, RbrSat03-75, and RbrSat08-176) and, because of this, they are not good markers for ancestry determination. Most A chromosomes share two satDNAs with the B chromosome. The A chromosomes that share more satDNAs with the B chromosome are pairs M7 and S11, three and four, respectively, being good candidates to be involved in the origin of B chromosome. Three satDNAs (RbrSat01-171, RbrSat03-75, and RbrSat08-176) shared between pair M7, S11, and the B chromosome are also located on other A chromosomes. Furthermore, the pair S11 harbors the satDNA RbrSat04-168 that is an exclusive sequence shared with the B chromosome. The pericentromeric region of pair S11 fits exactly the composition of pericentromeric region of the B chromosome, supporting the hypothesis of its involvement in B chromosome origin.

Some controversial ideas were proposed for the origin of B in *R. brasiliensis.* First, the authors of a previous paper [35] proposed the origin from one or several chromosomes, including, for example, the pair S11 as supported here by satDNA mapping. Pairs L2, L3, M5, and S11 harbor 5S rDNA clusters that are shared with the B chromosome [35] and could be involved in its origin. Second, the analysis made by the authors of a previous paper [14] did not support the autosomal origin hypothesis, based on the presence of 5S rDNA and H3 gene clusters in the B chromosome that are shared with most A chromosomes (including the X chromosome), except the pair S11. However, the occurrence of these repetitive DNAs in the B chromosome could be more related to transposition events after its origin than ancestry [14]. These data, including the individuals from distinct populations, support the multiregional origin of the B chromosome in *R. brasiliensis* or dynamics for repetitive DNA organization for both A and B chromosomes, causing the emergence of new B chromosome variants. Based on a cytomolecular analysis, multiple B chromosome variants were described, for example, in rye, *Secale cereale* [43], and in the grasshoppers *X. d. angulatus* [36] and *E. plorans* [44].

Although our findings strongly support the ancestry of the B chromosome from the pair S11 in *R. brasiliensis* (at least in Lagoa do Carro/PE population), we should also point attention to the pair M7 that share three satDNAs with the B chromosome. In other populations (including Lagoa do Carro/PE) the chromosome M7 harbors H3 histone gene, which in some individuals is exclusively shared with the B chromosome [14,45]. This suggests the involvement of M7, besides the pair S11, in B chromosome ancestry. It is similar to *L. migratoria* in which the B chromosome ancestry is putatively from two chromosomes, the pairs 8 and 9 [8], as in rye [46]. On the other hand, we should bear in mind that the pair M7 harbors one satDNA (RbrSat05-179) that is not observed in the B chromosome. Moreover, considering the high dynamism of the H3 histone gene (in number of clusters) in *R. brasiliensis*, it is possible that this gene was acquired later by the B chromosome. To shed light on this possibility, individuals from multiple populations should be studied using the distinct probes. 

The satDNA mapping in *S. rubiginosa* suggests an autosomal origin for B chromosome from the pair S9. This chromosome shares five satDNAs with the B chromosome, two of them exclusive for this chromosome (SruSat06-363 and SruSat07-232), thus supporting the ancestry of the B chromosome from this bivalent. Furthermore, the pair S9 also harbors other three satDNAs present in the B chromosome, SruSat02-170, SruSat03-170, and SruSat08-172. Interestingly, those two exclusive satDNAs in S9 also are abundant in the B chromosome. This means that these repeats were massively amplified covering almost the entire length of those two chromosomes. The SruSat01-194 that is present in the pair M6 is also highly abundant in the B chromosome. We ruled out the possibility of B origin from M6 due to the absence of other three satDNAs that are present in the B chromosome, including those pericentromeric satDNA. Furthermore, if the pair M6 is involved in B chromosome origin it has a secondary contribution in comparison to the pair S9. It should be noted that SruSat01-194 corresponds to the most abundant satDNA in the *S. rubiginosa* genome, visible in the B chromosome as a large block likely due to amplification after its origin. It might be possible that the presence of this repeat in other A chromosomes (including pair S9), but arranged non-tandemly, makes it difficult to reach the FISH threshold resolution.

The satDNA content and distribution in the B chromosome of *X. d. angulatus* indicate a more complex evolution than in *R. brasiliensis* and *S. rubiginosa*, with additional chromosomal rearrangements after the B chromosome origin followed by accumulation/deletion involving repeats, as suggested by the previous analyses [35,36] (see below). Even though there are 11 satDNAs clustered on the A chromosomes of *X. d. angulatus*, only three of them are present in the B chromosome. There is no satDNA exclusively shared between the B chromosome and one chromosome of A complement. However, the pair S10 shares the most satDNAs with B chromosome (three satDNAs families), and it seems to be the ancestral pair involved in the B chromosome origin. The three satDNAs shared between B chromosome and the pair S10 are located at pericentromeric or interstitial regions (not far from the centromere), highlighting the origin of the B chromosome from about the half proximal part of the pair S10. Recently, it was suggested that pericentromeric and proximal regions enriched of repetitive DNAs were involved with the B chromosome origin in *X. d. angulatus*, followed by repetitive DNA amplification and rearrangements, like inversions [36]. 

Although the origin of the B chromosome in *X. d. angulatus* from the proximal part of the pair S10 is supported by current data, the presence of two other satDNAs in the pericentromeric region of pair S10 (XanSat02-21 and XanSat12-246), not shared with the B chromosome, is contrary to this hypothesis. The difference between the satDNA content in B and chromosome S10 can be explained by the changes of satDNAs amounts in the B chromosome during its evolution. In that way, the satDNA XanSat03-10 was amplified in the pericentromeric region of the B chromosome, while the other ones were completely deleted or conserved in small copy number, not detected by FISH. Interestingly, XanSat03-10 is a unique satDNA exclusively located in the pericentromeric region of all A chromosomes, likely involved in centromeric function. This could be the explanation for its amplification in the centromere of B chromosome, giving more stability through cell divisions. Besides amplification/deletion of satDNAs, the distribution of repeats in the B chromosome suggests the possibility of putative events of duplication and inversion that gave origin to the terminal region. The amplification of satDNAs after its origin and the changing satDNA repeat abundance was postulated in *E. monticola* [7]. Moreover, the putative duplication and inversion on the B chromosome of *X. d. angulatus* highlights how dynamic the repetitive DNAs are on this element, leading to the emergence of distinct morphotypes.

## 5. Conclusions

The present data expands the knowledge about the B chromosomes composition and their origin in grasshoppers. Our results provide support for the intraspecific origin of the B chromosome in the three species, like in the other species of grasshoppers [7,16]. Although the B chromosomes share some meiotic peculiarities with the X chromosomes that suggested origin from this chromosome, the current knowledge indicates a more common origin from autosomes in grasshoppers [7,8,16,47]. Furthermore, the species studied here and other grasshopper species with B chromosome ancestry [7,8,47] support the recurrent involvement of small chromosomes in the B chromosome origin. This could be due to the fewer number of genes and the enrichment of repetitive DNAs in small autosomes [7,31,48]. The analysis of other populations employing the repetitive DNA markers used here will shed light on the evolution of B chromosome polymorphism in the species.

## Figures and Tables

**Table 1:** Characteristics of satellite DNA (satDNA) families isolated from the genomes of three grasshopper species, including their monomer sizes, base pair richness, and genome abundances.

Species	SatDNA Superfamily	SatDNA Family	Monomer Size (nt)	A+T (%)	Abundance (%)	Divergence (%)
*R. brasiliensis*	SF1	RbrSat01-171	171	59.3	0.766	4.98
	-	RbrSat02-410	410	47.9	0.224	8.94
	-	RbrSat03-36	36	44.5	0.126	8.45
	SF1	RbrSat04-168	168	59.4	0.105	18.14
	-	RbrSat05-179	179	59.1	0.061	4.96
	-	RbrSat06-165	165	59.2	0.056	1.23
	-	RbrSat07-240	240	60.4	0.047	11.56
	-	RbrSat08-176	176	58.0	0.042	6.27
	-	RbrSat09-238	238	63.9	0.025	8.75
	-	RbrSat10-268	268	62.7	0.021	7.79
	-	RbrSat11-233	233	58.0	0.016	7.85
	-	RbrSat12-180	180	57.8	0.010	2.06
Total					1.499	
*S. rubiginosa*	-	SruSat01-194	194	58.3	0.730	4.30
	-	SruSat02-170	170	54.4	0.476	4.64
	-	SruSat03-170	170	59.9	0.287	7.79
	-	SruSat04-301	301	48.9	0.244	4.77
	-	SruSat05-441	441	50.6	0.135	23.18
	-	SruSat06-363	363	57.1	0.126	9.61
	-	SruSat07-232	232	61.2	0.116	9.23
	-	SruSat08-172	172	58.2	0.032	16.24
	-	SruSat09-107	107	61.7	0.026	10.85
Total					2.172	
*X. d. angulatus*	-	XanSat01-8	8	62.5	0.627	4.62
	-	XanSat02-21	21	28.6	0.586	4.41
	-	XanSat03-10	10	60.0	0.464	9.38
	-	XanSat04-10	10	60.0	0.228	5.22
	SF1	XanSat05-267	267	56.7	0.087	5.50
	-	XanSat06-168	168	64.3	0.069	4.53
	SF1	XanSat07-279	279	60.2	0.053	11.08
	-	XanSat08-16	16	56.2	0.033	4.38
	SF2	XanSat09-130	130	63.1	0.024	10.61
	-	XanSat10-289	289	60.2	0.022	7.27
	-	XanSat11-51	51	47.1	0.019	3.97
	-	XanSat12-246	246	59.2	0.018	11.81
	-	XanSat13-281	281	56.3	0.018	4.61
	SF2	XanSat14-128	128	62.5	0.017	14.90
	-	XanSat15-228	228	59.7	0.017	5.18
	-	XanSat16-21	21	42.9	0.014	9.79
	-	XanSat17-15	15	53.4	0.013	11.56
	-	XanSat18-21	21	76.2	0.013	6.33
Total					2.322	

**Table 2:** Chromosome location of satDNAs in three grasshopper species. For each species at the bottom is indicated the number of satDNA families per chromosome and the amount of satDNAs shared with the B chromosome. p: pericentromeric, i: interstitial, d: distal, nc: nonclustered.

Species	SatDNA Family	Chromosome Location												
		1	2	3	4	5	6	7	8	9	10	11	X	B
*Rhammatocerus brasiliensis*	RbrSat01-171	p	p			p		p	p	p		p		p
	RbrSat02-410	nc												
	RbrSat03-36	p	p	p	p	p	p	p	p	p	p	p	p	p
	RbrSat04-168											p		p
	RbrSat05-179							p		p				
	RbrSat06-165	nc												
	RbrSat07-240	nc												
	RbrSat08-176			p	p		p,i	p			p	p	i	p
	RbrSat09-238									i				
	RbrSat10-268	nc												
	RbrSat11-233	nc												
	RbrSat12-180	nc												
Total		2	2	2	2	2	2	4	2	4	2	4	2	4
shared with B		2	2	2	2	2	2	3	2	2	2	4	2	
*Schistocerca rubiginosa*	SruSat01-194						d							i
	SruSat02-170	p	p	p	p	p	p	p	p	p	p	p	p	i
	SruSat03-170	p,d		p,d		p			p	p	p	p		i
	SruSat04-301	nc												
	SruSat05-441	nc												
	SruSat06-363									i,d				2i
	SruSat07-232									i,d				i
	SruSat08-172			p	i	p	p			p	p,i	p		p
	SruSat09-207	nc												
Total		2	1	3	2	3	3	1	2	5	3	3	1	6
shared with B		2	1	3	2	3	3	1	2	5	3	3	1	
*Xylleus discoideus angulatus*	XanSat01-8	i	i	d	i	d	i	d	d	d	d	d	2i	
	XanSat02-21	p	p	p	p	p	p	p	p	p	p,d	p	p	
	XanSat03-10	p	p	p	p	p	p	p	p	p	p	p	p	p,i,d
	XanSat04-10			p			p	p						
	XanSat05-267						p				p,i			2i
	XanSat06-168					p	i							
	XanSat07-279	i	d				p							
	XanSat08-16						d							
	XanSat09-130	nc												
	XanSat10-289	nc												
	XanSat11-51		d											
	XanSat12-246	p	p	p	p	p	p	p		p	p	p		
	XanSat13-281							p		d	i			i,d
	XanSat14-128	nc												
	XanSat15-228	nc												
	XanSat16-21	nc												
	XanSat17-15	nc												
	XanSat18-21	nc												
Total		5	6	5	4	5	9	6	3	5	6	4	3	3
shared with B		1	1	1	1	1	2	2	1	2	3	1	1	

## Supplementary Materials

The following are available online at http://www.mdpi.com/2073-4425/9/11/523/s1, Table S1. Primers designed in this work and used for PCR amplification of satellite DNAs in the three species of grasshoppers.
