# Supplementary material for: Satellite DNAs Unveil Clues about the Ancestry and Composition of B Chromosomes in Three Grasshopper Species
Source: Genes (Basel). 2018 Oct 26;9(11):523. doi: 10.3390/genes9110523 (PMC6265867; doi:10.3390/genes9110523)
Supplement: Supplementary file 1 [file genes-09-00523-s001.pdf]

Article

Satellite DNAs unveil clues about the ancestry and composition of B chromosomes in three grasshopper species

Diogo Milani, Vanessa B Bardella, Ana BSM Ferretti, Octavio M Palacios-Gimenez, Adriana de S Melo, Rita de C de Moura, Vilma Loreto, Hojun Song, Diogo C Cabral-de-Mello*

Supplementary table

Table S1. Primers designed in this work and used for PCR amplification of satellite DNAs in the three species of grasshoppers.

Species	SatDNA	Primer F	Primer R
R. brasiliensis	RbrSat01-171	5’ TGTTATTTAGTGTTCCACACC 3’	5’ TTCAATAAACACACTGAAAGTGT 3’
	RbrSat02-410	5’ GAGGACATATGAGAATTTGGG 3’	5’ CTACTTTGGCCATTACACTTC 3’
	RbrSat03-75	5’ TTGCTAGGACAGGGACTCAA 3’	5’ GCAGGAGGCTATGGGTTTG 3’
	RbrSat04-168	5’ GCTCCACAGAAAAGCACCAT 3’	5’ CCCCAGACTAGTTTTGACGT 3’
	RbrSat05-179	5’ TCCGCACTGTAATAATTCAAAT 3’	5’ ATCACTGGCAAAGGGTGACT 3’
	RbrSat06-165	5’ ACCTCAAATCGATCTCCAGAT 3’	5’ ACAACATAGCTGCTACTTCTG 3’
	RbrSat07-240	5’ CAGTGGCGAATGATTTCTACA 3’	5’ GACTCCTCGTAGTGTGGAG 3’
	RbrSat08-176	5’ GTGCGCCACGCCTAATTTC 3’	5’ GGTCCCGCAAAGTTTCTGC 3’
	RbrSat09-238	5’ TTCCATCTTCGTGTTACCTG 3’	5’ ACAGCAGCGATTTATTTGGG 3’
	RbrSat10-268	5’ TTAAACGTGGCCTAACTGTC 3’	5’ CGTTGAAGGCGTTAAACAAG 3’
	RbrSat11-233	5’ AGGAGAGAAGACACCGCCA 3’	5’ CAGGCTCCCTCTGCAAAATA 3’
	RbrSat12-180	5’ AGTGTCTGGCAGACCATACA 3’	5’ ACGTGTACCCAGACTTCCAA 3’
S. rubiginosa	SruSat01-194	5’ TAGCAAAACCGCAGAAAACCT 3’	5’ CGCATTACCACTTCTCGCTT 3’
	SruSat02-170	5’ TCGTCATTAAGAGGCTCTGA 3’	5’ TGGAACACACAAATTGCGTC 3’
	SruSat03-170	5’ GCACTATTCCTTCTTTACTGC 3’	5’ ATCGGAAAGGAGATACTCGTA 3’
	SruSat04-301	5’ CTGCCAGGAAGTTTCATATC 3’	5’ ATTAAAACTGTGTGCCGGAC 3’
	SruSat05-441	5’ TGCCATGTAAGCAGGAGAT 3’	5’ GACGCTCTATCCATCTGAG 3’
	SruSat06-363	5’ GAGGCAAAAGCGCACTCAAA 3’	5’ GGAAGGTAAGGGCGTATCG 3’
	SruSat07-232	5’ AATGTTGCAACCACACACAC 3’	5’ ACGCTCCCATTGAGTAAATG 3’
	SruSat08-172	5’ TGTACTTCAGTCGGCATTGT 3’	5’ AGAAGACTGAGTCTCCTTAC 3’
	SruSat09-107	5’ ACACTGCTGAAAGCTTCCTTA 3’	5’ CTTCCAGTCCACGTTCACAT 3’
X. d. angulatus	XanSat01-8	5’ CGAAAAGTCGAAAAGTCGA 3’	5’ ACTTTTCGACTTTTCGACTT 3’
	XanSat02-21	5’ GGCTATCTTCCCGCACCG 3’	5’ GGCCGGTGCGGGAAGATA 3’
	XanSat03-10	5’ CAGCCAAAATCAGCCAAAAT 3’	5’ ATTTTGGCTGATTTTGGCTG 3’
	XanSat04-10	5’ TTCGGCAAACTTCGGCAAA 3’	5’ GTTTGCCGAAGTTTGCCGA 3’
	XanSat05-267	5’ CATCCTCCTACCAGATGG 3’	5’ AAGACTGCTGCAGGGCAA 3’
	XanSat06-168	5’ CCGTTACAGAACAACATAAAT 3’	5’ ATATCAAACTCTCAGCCAGT 3’
	XanSat07-279	5’ CTTACTACATGAAGACTGAC 3’	5’ ATATCCTTTGGTCACACAAG 3’
	XanSat08-16	5’ GTCTCCGCATTTTCTTGTCT 3’	5’ AAGAAAATGCGGAGACAAGA 3’
	XanSat09-130	5’ CAATGACCATGTAGAAGGCT 3’	5’ GCAACATCAAGAGGAATCAG 3’
	XanSat10-289	5’ TCCGACAGGGTGGATTTAAA 3’	5’ GGAAATGCGTTGTCCTTATA 3’

XanSat11-51	5' GGAGTGATTCTTAGTGACTG 3'	5' CCTTGCCACCCAGTATCAA 3'
XanSat12-246	5' TCAGTAGTAAGAGAGCAATAA 3'	5' CACATCATCATAAAAGTGCA 3'
XanSat13-281	5' GATTATGGCCAGTGTCCATA 3'	5' GATTTCCACCATAAATAGAGT 3'
XanSat14-128	5' AAAACGTGTTCTCTGCTGTGT 3'	5' CATATTCCTCGTGATGATTTC 3'
XanSat15-228	5' AGCTAGACCACAGTAACTAC 3'	5' TTACGCTCAGTTAGTACCGA 3'
XanSat16-21	5' CGAGTTACGCAAAACGTGG 3'	5' CCCCACGTTTTGCGTAACT 3'
XanSat17-15	5' TAATCTCCGCACTCATAATCT 3'	5' TGAGTGCGGAGATTATGAGT 3'
XanSat18-21	5' TGGAAAAACGTCAATATTTT 3'	5' AAAAATATTGACGTTTTTCC 3'
